# Bone Density Testing: An Under-Utilised and Under-Researched Health Education Tool for Osteoporosis Prevention?

**DOI:** 10.3390/nu2090985

**Published:** 2010-09-16

**Authors:** Tania Winzenberg, Brian Oldenburg, Graeme Jones

**Affiliations:** 1 Menzies Research Institute, Private Bag 23, Hobart TAS 7001, Australia; Email: graeme.jones@utas.edu.au; 2 International Public Health Unit, Department of Epidemiology and Preventive Medicine, Monash University, Alfred Hospital, Melbourne 3004, Australia; Email: brian.oldenburg@med.monash.edu.au

**Keywords:** osteoporosis, prevention, calcium, physical activity, bone density

## Abstract

Feedback of fracture risk based on bone mineral density (BMD) is an under-explored potential osteoporosis education intervention. We performed a randomised controlled trial of either an osteoporosis information leaflet or small group education (the Osteoporosis Prevention and Self-Management Course (OPSMC)), combined with individualised fracture risk feedback in premenopausal women over two years. Women with a mean T-score at spine and hip of <0 were informed they were at higher risk of fracture in later life and those with T-score ≥ 0 were informed they were not. Women receiving feedback of high fracture risk had a greater increase in femoral neck, but not lumbar spine, BMD compared to the low risk group (1.6% p.a. *vs*. 0.7% p.a., p = 0.0001). Participation in the OPSMC had no greater effect on BMD than receiving the leaflet. Femoral neck BMD change was associated with starting calcium supplements (1.3% p.a., 95% CI +0.49, +2.17) and self-reported physical activity change (0.7% p.a., 95% CI +0.22, +1.22). Mother’s report of increasing their children’s calcium intake was associated with receiving the OPSMC (OR 2.3, 95% CI 1.4, 3.8) and feedback of high fracture risk (OR 2.0, 95% CI 1.2, 3.3). Fracture risk feedback based on BMD could potentially make an important contribution to osteoporosis prevention but confirmation of long-term benefits and cost effectiveness is needed before implementation can be recommended.

## 1. Introduction

Osteoporosis is commonly encountered in general practice (0.9 per 100 patient encounters each for fractures and for osteoporosis) [1] and is included in the National Health Priority area of musculoskeletal disorders because of its public health importance. In Australia, the health costs of osteoporotic fractures are estimated at $7.5 billion [2]. At an individual level, death, loss of ability to live independently and long-term restrictions on usual activities are all common sequelae of osteoporotic fracture [3]. Its prevention is therefore of great importance and finding effective ways to intervene for prevention is a high priority.

Bone mineral density (BMD) is a major predictor of osteoporotic fracture [4,5]. Peak bone mass (the maximum bone mass attained in a person’s life) and the rate of subsequent bone loss [6] are both important determinants of BMD in later life. In fact, premenopausal bone mass is as important as post-menopausal bone loss for prediction of fracture [6]. Peak bone mass may not be reached until well into adult life [7] and a substantial amount of bone loss occurs prior to menopause through age-related bone loss [8]. Thus in premenopausal women it is potentially possible to intervene to both improve peak bone mass and slow age-related bone loss, and through this maintain adequate BMD and reduce fracture risk in old age.

Bone density can be improved in premenopausal women. For example, a meta-analysis of trials of calcium supplementation in premenopausal women gave an effect size of 1.3% per annum across a combination of sites [9] for supplements and in a meta-analysis of controlled trials, exercise training programs prevented or reversed almost 1% of bone loss per annum at both the lumbar spine and femoral neck [10]. However, few studies have examined how to intervene in younger women to induce the behaviour changes needed to improve BMD.

Risk communication interventions for behaviour change are effective across a range of clinical areas and settings [11]. Individualised risk estimation is a key factor in increasing their efficacy [12]. Fracture risk assessment and feedback is increasingly recognised as having a potential role in prevention and management of osteoporosis [13] but this remains under-investigated. A key component of fracture risk assessment is bone mineral density testing. Bone density testing is common. For example, over 154,000 Medicare rebatable bone density tests were performed in Australia alone in 2005 and this number is increasing [14]. This represents an enormous opportunity for fracture prevention if there were a simple way to link bone density results with fracture risk feedback to patients to promote lifestyle behaviour change to improve bone density. Three uncontrolled studies have suggested that BMD screening with feedback of results combined with an information leaflet increases self-reported osteoporosis preventive behavior change at twelve months, in women aged 30 to 80 years [15] and in exclusively premenopausal women [16,17]. In particular, greater changes were reported in women with low BMD. However, there have been no longer-term follow-up studies or studies measuring effects on BMD. Our study aimed to determine the effectiveness of individualised fracture risk based on bone density for improving osteoporosis preventive behaviours and bone density in premenopausal women.

## 2. Experimental Section

The detailed methods of the study have been previously published [18,19]. In brief, this was a randomized controlled trial of two osteoporosis educational interventions in combination with individualised fracture risk feedback based on bone density. The study was performed in 2000–2003 in a mainly Caucasian population in Southern Tasmania. Women aged 25–44 years were randomly selected from the year 2000 Tasmanian Electoral Roll. Women were excluded if they had previously had measurement of bone densitometry, had thyroid disease, renal failure, malignancy or rheumatoid arthritis, had a history of hysterectomy, were on hormone replacement therapy or were pregnant or planning pregnancy within 2 years of study entry, or were lactating (total excluded = 146). Ethics approval was obtained from the Royal Hobart Hospital Ethics Committee and all participants gave written informed consent.

### 2.1. Interventions

The effect of fracture risk feedback based on bone density was studied in combination with osteoporosis education. 

#### 2.1.1. Bone density feedback

We measured participants BMD at the spine and hip at baseline (Hologic QDR2000, Waltham, MA). Those with a mean T-score at spine and hip of greater than or equal to 0 received a letter informing them that they were not at a higher risk of fracture in later life (normal risk group), whereas those who had a mean T-score of less than 0 were informed that they were at higher risk (high risk group). This approach was based on the three-fold higher risk of fracture observed both in later life and in the early postmenopausal period [5] for those in the lower half of the BMD distribution. 

#### 2.1.2. Education

Before participants’ BMD results were known, they were randomised using computer generated random numbers to receive either an information leaflet produced by Osteoporosis Australia (“Understanding Osteoporosis”) or the Osteoporosis Prevention and Self-management Course (OPSMC). The OPSMC is a chronic disease self-management course developed by the Arthritis Foundation of Victoria and utilized by Osteoporosis Australia, aiming to increase knowledge, improve confidence and awareness and self-management of osteoporosis prevention with an emphasis on promoting appropriate lifestyle change such as increasing calcium intake, increasing appropriate physical activity and smoking cessation. It is a small group program, with a maximum group size of 16, consisting of four 2-hour sessions held weekly delivered by 2 of 12 Department of Health and Human Services allied health professionals (physiotherapists, occupational therapists and nurses). Sessions included lectures, discussion, brainstorming, demonstration and small group work. Participants randomised to the leaflet intervention received the Understanding Osteoporosis leaflet providing a description of osteoporosis, an overview of the role of lifestyle factors such as diet, exercise and smoking and outlines ideal levels of calcium intake and exercise. 

### 2.2. Outcome Measures

Primary outcome measures were BMD, calcium intake and physical activity and fitness. We measured BMD at the femoral neck and lumbar spine by Hologic QDR2000 densitometer on fan beam setting at baseline and 2 years. Reproducibility in adults is of the order of 2–3% [5]. Usual calcium intake was assessed yearly by a validated short food frequency questionnaire (FFQ) designed specifically to measure calcium intake [20]. Information on whether participants were taking calcium supplements was also obtained by questionnaire, with participants classified as taking calcium supplements if they reported taking a supplement containing calcium alone or as a main ingredient at least 4 times per week. Energy expenditure and sports participation was assessed annually by a questionnaire validated in US adolescents [21] which we have previously used in Tasmanian women of this age group in whom it was associated with bone mass at the femoral neck [22]. This questionnaire measured strenuous physical activity levels by how many days in the last 14 the participants reported performing at least 20 minutes of strenuous exercise in five categories ((1) 0 days, (2) 1–2 days, (3) 3–5 days, (4) 6–8 days, (5) 9 or more days). Muscle strength and endurance fitness was assessed at baseline and two years. Muscle strength was assessed by dynamometry in the lower limb. Endurance fitness was assessed by bicycle ergometer where physical work capacity at 170 beats per minute was estimated by progressively increasing sub maximal workloads [23]. 

As secondary outcomes we assessed maternal report of changing their children’s calcium intake and/or physical activity by questionnaire. At 1 year, participants were asked: “If you have children, have you changed their: Calcium intake? (yes/no) Physical activity? (yes/no)”. At 2 years, they were asked: “If you have children, in the last year have you changed their: Calcium intake? Physical activity?” with options of increased, decreased or same.

### 2.3. Other Factors

Other factors measured at baseline and 2 years included: height and weight with body mass index calculated (kg/m^2^) and questionnaire assessments of smoking history (current/former/never, cigarettes per day, age at uptake, age at ceasing), breastfeeding history (ever breastfed, time since last breastfeeding), number of children, family history of osteoporosis and/or fracture, as well as fracture history in the subject, education level (4 point scale: less than grade 10, up to grade 10, completed grade12, tertiary), employment status of main financial provider in the household (employed or unemployed), hours of employment of the respondent (0, less than or equal to 20 or >20 hours per week) and marital status. 

At one and two years, participants were also asked asking a series of yes/no questions (by mailed out questionnaire) to assess self-reported change in smoking, dietary calcium intake, calcium supplement use and physical activity.

### 2.4. Statistical Analysis

Simple linear regression and one-way ANOVA were used for continuous and categorical measures respectively to examine the relationships between BMD change and intervention groupings, and changes in osteoporosis preventive behaviors. Multiple regression modeling, including potential confounders, was then used to examine the relationships between risk feedback group and education interventions and BMD change as well as changes in behavior and BMD change. Three sets of analyses were performed:

(1) available data analysis, *i.e.*, including all participants who reached 2 years of follow-up;
(2) intention to treat, in which we imputed missing data at 2 years using the method of last observation carried forward [24] for measured variables, and imputed no change for self-reported behavioral change variables; and
(3) per protocol analysis defined in two ways: firstly, by whether subjects attended at least one educational session of the OPSMC; and secondly, by whether they attended all four OPSMC sessions.

A sensitivity analysis was also performed omitting subjects with a baseline T-score < −2.5, which may lead to pharmacological treatment for osteoporosis in our location. 

For secondary outcomes, intention to treat analysis was performed using logistic regression to determine factors associated with maternal report of children’s calcium intake and physical activity change. 

All analyses were performed in Stata version 7 (Stata Corporation, Texas, USA). Statistical significance was set as p < 0.05 (two-tailed).

## 3. Results and Discussion

### 3.1. Results: Primary Outcomes—BMD and Behaviour Change

Of 470 women recruited (response rate of 64%), 415 (88%) reached final follow-up at 2 years. There were no statistically significant differences in baseline demographics and proportions of participants receiving the OPSMC and high risk (low T-score) feedback between those completing the study and those withdrawing (data not shown). As expected, at baseline women in the high risk category were shorter and lighter than those in the normal risk category. There was a trend (p = 0.05) for a greater proportion of women in the high risk group to be taking calcium supplements, but the proportion in both groups was small. The groups were otherwise similar. Only three subjects had a femoral neck or lumbar spine T-score of less than −2.5.

Table 1 summarises the effects of each intervention and of behaviour changes on femoral neck and lumbar spine BMD. Participants who received feedback of high risk had a higher percentage rate of change in femoral neck BMD than those who received feedback of normal risk. There was no difference in the rate of change in femoral neck BMD between the leaflet and OPSMC groups. There were no differences in rates of lumbar spine BMD change between either risk groups or education groups. 

**Table 1:** Effects of interventions (risk feedback and education) and behavior changes on bone density at the femoral neck and lumbar spine (adapted from Winzenberg 2006 [19]).

	Multivariate β ^a,b ^(95% CI)	
**Femoral Neck BMD change (% p.a.)**		
*Intervention group*		
High *vs.* normal risk feedback	**+0.86**	**(+0.39, +1.34)**
OPSMC v leaflet	+0.14	(−0.32, +0.62)
*Behaviour change*		
Commenced calcium supplements	**+1.33**	**(+0.49, +2.17)**
Calcium intake change (per 100 mg)	−0.03	(−0.08, +0.01)
Persistent smoking cessation	−0.04	(−1.16, +1.08)
Persistent self-reported physical activity change	**+0.72**	**(+0.22, +1.22)**
Persistent increase in strenuous activity	+0.11	(−0.45, +0.67)
Change in work capacity (per W)	−0.06	(−0.48, +0.36)
Change in leg strength (per SD)	+0.02	(−0.22, +0.26)
**Lumbar Spine BMD change (% p.a.)**		
*Intervention group*		
High *vs.* normal risk feedback	−0.01	(−0.32, +0.30)
OPSMC v leaflet	+0.09	(−0.21, +0.40)
*Behaviour change*		
Commenced calcium supplements	+0.18	(−0.37, +0.73)
Calcium intake change (per 100 mg)	−0.01	(−0.04, +0.02)
Persistent smoking cessation	+0.11	(−0.62, +0.85)
Persistent self-reported physical activity change	−0.05	(−0.38, +0.28)
Persistent increase in strenuous activity	−0.16	(−0.53, +0.21)
Change in work capacity (per W)	**+0.31**	**(+0.03, +0.59)**
Change in leg strength (per SD)	+0.05	(−0.10, +0.22)

^a^ adjusted for age, and difference in weight and height between baseline and 2 years. For each intervention, results are also adjusted for the other intervention. For behavior change variables, each is adjusted for the other behaviour change variables. **Bold** denotes statistical significance. ^b^ available data analysis.

**Figure 1:**
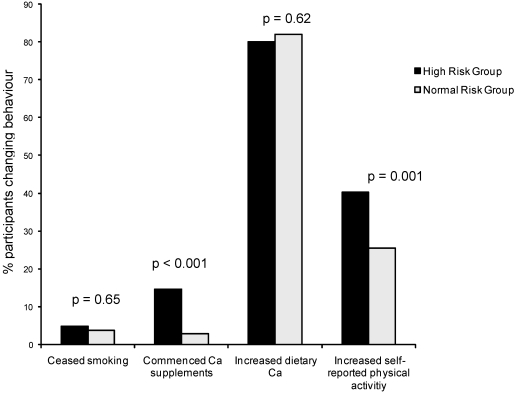
Effect of high risk feedback on osteoporosis preventive behavior change (adapted from Winzenberg, 2006 [19]).

Figure 1 shows differences in osteoporosis preventive behaviors at two years by risk feedback group. A greater proportion of subjects in the high risk feedback group commenced taking calcium supplements (as measured by FFQ) and reported changes in physical activity than in the high T-score group. Levels of dietary calcium intake and smoking cessation (Figure 1) and strenuous activity levels, average leg strength change, and change in work capacity (data not shown) were similar across risk feedback groups. There were no differences in any behaviour change variable between education groups (data not shown). 

There were positive associations between change in femoral neck BMD and calcium supplement use (whether measured by FFQ or self-reported behavior change) and physical activity (by self-report but not questionnaire assessment) (Table 1). At the lumbar spine, there was a positive association between change in work capacity and lumbar spine BMD change but no associations with other behaviour change measures. 

These associations were unchanged using intention to treat analysis and per protocol analysis or if participants with a baseline T-score of less than or equal to −2.5 were omitted (data not shown).

### 3.2. Results: Secondary Outcomes—Maternal Report of Changing Children’s Behaviour

This sub-analysis was performed in the 354 participants who were mothers, as previously described [25]. Baseline characteristics of the mothers were similar between intervention groups (data not shown). Nine percent of mothers commenced calcium supplements themselves and 32% reported changing their level of physical activity. 

The proportion of mothers reporting increasing their children’s calcium intake in the two risk feedback groups was similar after 1 year (54% *vs.* 46% for high *vs.* normal risk groups respectively, p = 0.13), but was higher in the high risk group after 2 years (58% *vs.* 39%, p = 0.001). Women receiving the OPSMC more often reported increasing their children’s calcium intake at both time points (59% *vs.* 42% at year 1, p = 0.002 and 56% *vs.* 41% at year 2, p = 0.006). There was no association between risk feedback group or education type and maternal report of improving their children’s physical activity (data not shown). 

Predictors of women reporting changing their children’s calcium intake and physical activity at 2 years are given in Table 2. Having a child under the age of 18 years old, receiving feedback of high fracture risk, receiving the OPSMC and the mother’s own behaviour change during the study were positively associated with maternal-report of changing in children’s calcium intake at 2 years. In contrast, the only factor associated with maternal report of change in children’s physical activity was the mother’s own self-reported physical activity change. 

**Table 2:** Predictors of maternal report of changing children’s calcium intake and physical activity (adapted from Winzenberg 2006 [25]).

	Univariable OR (95% CI)	Multivariable ^a^ OR (95% CI)
Predictors of calcium intake increase		
Youngest child <18 yrs old	**4.30 (1.20, 15.42)**	**4.32 (1.11, 16.75)**
T-score group	**2.16 (1.38, 3.41)**	**1.97 (1.19, 3.27)**
OPSMC	**1.88 (1.20, 2.95)**	**2.28 (1.37, 3.80)**
Mother commenced Ca supplements (2-years)	**3.38 (1.39, 8.26)**	2.55 (0.97, 6.72) ^b ^
Mother Increased physical activity	**2.70 (1.68, 4.36)**	**2.21 (1.32, 3.72)**
Predictors of physical activity increase		
Youngest child <18 yrs old	2.65 (0.72, 9.74)	1.58 (0.38, 6.68)
T-score group	0.94 (0.57, 1.55)	0.87 (0.48, 1.57)
OPSMC	1.07 (0.65, 1.78)	1.12 (0.63, 2.01)
Mother commenced Ca supplements (2-years)	1.03 (0.38, 2.75)	0.79 (0.26, 2.86)
Mother Increased physical activity	2.41 (1.42, 4.10)	**2.74 (1.51, 4.98)**

^a^ Adjusted for other items in table, education level, marital status, family and personal history of fracture, employment status of main financial provider in household and age. **Bold** denotes statistical significance. ^b^ p = 0.056.

### 3.3. Discussion

This study provides the best evidence to date to that individualised fracture risk feedback can improve rates of calcium supplement, physical activity and most importantly, bone mineral density, even in a younger population of women. Moreover, the fact that this occurred with the provision of a very simple leaflet educational intervention, with no added benefit from more complex group education is a very important point from the point of view of future implementation, as a simple leaflet would be cheap and easy to include with bone density results and risk feedback, compared to delivery of the OPSMC.

The BMD changes associated with changes in calcium supplement use and self-reported physical activity increases are of similar magnitude to those seen in randomised controlled trials of exercise and calcium supplements [9,10]. The ability to achieve these changes with relatively simple interventions has major potential public health benefits for osteoporosis prevention and fracture reduction in later life. For example, in drug trials a change in BMD of 5% with bisphosphonates leads to a 50% decrease in fracture risk [26] while regular walking is associated with a 50% decrease in hip fracture risk [27]. The behaviour changes could also contribute to the prevention of other chronic diseases such as cardiovascular disease, obesity and diabetes mellitus. However, confirmation of long-term benefits and assessment of the cost effectiveness of the intervention needs to occur before any recommendation for implementation at a population level is made. 

The differences we observed in associations between BMD changes and different physical activity measures at different BMD measurement sites is consistent with other literature. It has been reported that in premenopausal women, exercise interventions can change femoral neck BMD without muscle strength increases occurring concurrently [28]. Different types of exercise can also cause different BMD responses in the lumbar spine and femoral neck BMD [29,30]. The different aspects of physical activity captured in our different measures and the site dependency of the BMD effects of different activities may account for the variability in associations. It is also possible that the femoral neck may be more susceptible to lifestyle interventions in premenopausal women than the lumbar spine. 

The data we present on the secondary outcomes of maternal report of changing children’s behaviour must be interpreted cautiously, as the data come from subjective measures relying on maternal report. We further investigated this issue with a qualitative study interviewing 39 of these mothers [31]. In these interviews, mothers described a number of specific dietary changes they made to increase their children’s calcium intake as well as strategies for improving calcium intake and physical activity. These included approaches such as role modelling, providing information and ensuring their children were aware of the importance of calcium; having calcium-rich foods accessible for their children; working around children’s likes and dislikes. The detailed strategies described by the mothers provide some further support to suggest that the maternally reported changes are in fact real. If this is the case, fracture risk feedback could have health benefits not just for the individual woman receiving it, but also for their family as a whole. 

This study has several limitations. The number of women who persistently ceased smoking over the study period was small (n = 20), so the study had insufficient power to detect a BMD differences due to smoking cessation. While the 2-year follow-up period we implemented is substantial in comparison to behavioural intervention studies for osteoporosis, the effects we observed will need to persist in the longer-term if osteoporotic fractures are to be reduced as a result. Further follow-up of these women, who are now over a decade post-intervention, would be extremely valuable. For ethical reasons, we did not have an intervention group receiving BMD feedback alone without any other educational intervention. Previous studies of BMD feedback to change behaviour had included leaflet information [15,16,17] and there was therefore an argument that supplying such a leaflet would be the minimum intervention that would be ethically acceptable. A randomized controlled trial of written information alone found no changes in behaviour from the intervention [32] and we demonstrated substantial variation in effects between risk feedback groups. Thus while we cannot state with complete certainty which effects were due to risk feedback and which the leaflet, the data suggest that risk feedback was the more important component of the intervention. Lastly, while our data suggest that there may be positive effects of providing fracture risk feedback to mothers on their children; this requires confirmation by studies using objective measures of behaviour and bone density in their children.

## 4. Conclusions

Providing young women with an individualised assessment of their fracture risk combined with a simple educational leaflet results in behavioural changes leading to increases in hip but not lumbar spine bone density. These behaviour changes are potentially important for both osteoporosis prevention and the prevention of other common chronic diseases. Furthermore, these benefits may extend to these women’s families. The potential public health benefits of harnessing the prevention potential of the many bone density tests done each year worldwide are enormous. Further research to provide the evidence of long-term benefit and cost-effectiveness needed to support widespread implementation is therefore urgently needed. 
